# Distinct resting-state functional connections associated with episodic and visuospatial memory in older adults

**DOI:** 10.1016/j.neuroimage.2017.07.049

**Published:** 2017-10-01

**Authors:** Sana Suri, Anya Topiwala, Nicola Filippini, Enikő Zsoldos, Abda Mahmood, Claire E. Sexton, Archana Singh-Manoux, Mika Kivimäki, Clare E. Mackay, Stephen Smith, Klaus P. Ebmeier

**Affiliations:** a Department of Psychiatry, University of Oxford, Oxford, OX3 7JX, United Kingdom; b Centre for Research in Epidemiology and Population Health, INSERM, U1018, Villejuif, France; c Department of Epidemiology and Public Health, University College London, UK; d Functional Magnetic Resonance Imaging of the Brain Centre, University of Oxford, Oxford, OX3 9DU, United Kingdom

**Keywords:** Connectomics, Network modelling, Verbal memory, Visuospatial memory, Resting-state fMRI, Hippocampus

## Abstract

Episodic and spatial memory are commonly impaired in ageing and Alzheimer's disease. Volumetric and task-based functional magnetic resonance imaging (fMRI) studies suggest a preferential involvement of the medial temporal lobe (MTL), particularly the hippocampus, in episodic and spatial memory processing. The present study examined how these two memory types were related in terms of their associated resting-state functional architecture. 3T multiband resting state fMRI scans from 497 participants (60–82 years old) of the cross-sectional Whitehall II Imaging sub-study were analysed using an unbiased, data-driven network-modelling technique (FSLNets). Factor analysis was performed on the cognitive battery; the Hopkins Verbal Learning test and Rey-Osterreith Complex Figure test factors were used to assess verbal and visuospatial memory respectively. We present a map of the macroscopic functional connectome for the Whitehall II Imaging sub-study, comprising 58 functionally distinct nodes clustered into five major resting-state networks. Within this map we identified distinct functional connections associated with verbal and visuospatial memory. Functional anticorrelation between the hippocampal formation and the frontal pole was significantly associated with better verbal memory in an age-dependent manner. In contrast, hippocampus–motor and parietal–motor functional connections were associated with visuospatial memory independently of age. These relationships were not driven by grey matter volume and were unique to the respective memory domain. Our findings provide new insights into current models of brain-behaviour interactions, and suggest that while both episodic and visuospatial memory engage MTL nodes of the default mode network, the two memory domains differ in terms of the associated functional connections between the MTL and other resting-state brain networks.

## Highlights

• Episodic and visuospatial memory engaged a common medial temporal lobe substrate at rest.
• However, the resting-state functional connections of the MTL differed based on the memory demand.
• Visuospatial memory was associated with hippocampal-parietal and motorparietal interaction.
• Verbal memory was associated with hippocampus-frontal pole anticorrelation.
• Findings provide novel insights into resting-state brain-behaviour interactions in older adults.

## 1 Introduction

It is estimated that by 2050, nearly one in four people worldwide will be over 60, with older adults outnumbering children under 14 (WPA, 2015). In a rapidly ageing society, it is becoming increasingly important to understand the biological underpinnings of cognitive function in older age. Decline in episodic memory is often the first clinical presentation in patients with Alzheimer's disease (AD) and amnestic mild cognitive impairment, and the early stages of AD are also characterised by deficits in visuospatial memory (Lange et al., 2002, Serino et al., 2015). Medial temporal lobe structures, particularly the hippocampus, contribute to the processing of episodic memory as well as the representation of spatial information in the brain (Kumaran and Maguire, 2005, Nadel and Moscovitch, 1997, O'Keefe and Nadel, 1978). Reductions in hippocampal volume and impairments in its structural connections to other brain regions are implicated in the episodic and spatial memory deterioration commonly observed in ageing and dementia (Delbeuck et al., 2003, Jack et al., 2004, Serino and Riva, 2014).

Functional magnetic resonance imaging (fMRI) studies have furthered our understanding of the hippocampus' complex role in supporting episodic and spatial memory. Task-based fMRI studies suggest a preferential involvement of hippocampal activity during episodic and spatial memory tasks (Hirshhorn et al., 2012, Nadel et al., 2013, Robin et al., 2015, Ryan et al., 2009), but they tell us little about its relevant functional connections, which are usually studied using resting-state fMRI. In the resting-state brain, *i.e.* in the absence of a task, spontaneous activity within the hippocampus is synchronised (functionally connected) with a network of brain regions that together make up the default mode network (DMN). This resting-state network comprises the anterior and posterior cingulate cortices, precuneus, lateral temporal cortex, ventromedial prefrontal cortex, inferior parietal lobule and medial temporal lobe structures (Buckner et al., 2008, Raichle et al., 2001). The DMN is typically engaged at rest and during internally oriented tasks involving autobiographical memory, and is deactivated during cognitively challenging or externally oriented tasks (Andrews-Hanna et al., 2010a, Buckner, 2010, Spreng et al., 2009). Reduced functional connectivity (FC) of the DMN has been linked to memory impairment in AD patients and, not surprisingly, this network is the most frequently studied resting-state network in the context of memory decline. (Allen et al., 2007, Andrews-Hanna et al., 2007, Damoiseaux et al., 2008, Ferreira and Busatto, 2013, Hafkemeijer et al., 2012, Mevel et al., 2011, Wu et al., 2011).

Recent evidence suggests that the functional association of DMN with the hippocampus may vary based on cognitive demands. For instance, studies find synchronised activity between the hippocampus and DMN during episodic memory retrieval but not encoding, and between hippocampus and prefrontal networks during episodic but not spatial memory tasks (Andrews-Hanna et al., 2010b, Huijbers et al., 2011, Robin et al., 2015). Moreover, it is not simply functional connectivity (FC) *within* the DMN but its anticorrelations (negative correlation) with other organized resting-state networks like the task-positive dorsal attention network and central executive network (CEN) that may be essential for supporting cognition (Nekovarova et al., 2014, Onoda et al., 2012, Spreng et al., 2016, Spreng et al., 2010, Sridharan et al., 2008, Uddin et al., 2009). Graph-theory and whole-brain rs-fMRI based connectomics approaches allow us to expand on the traditional, more focused seed-based or single-network analyses, and examine these intra- and inter-network connections in greater detail (Cole et al., 2010, Smith et al., 2013, van den Heuvel and Sporns, 2013). Such network modelling methods map the “functional connectome” by parcellating the rs-fMRI data into a large number of small distinct brain regions (*nodes*) using (for example) high-dimensionality independent component analysis (ICA), and subsequently estimating the FC (*edges*) as the temporal correlations of node activity. These techniques have provided valuable insights into the organisation of the brain at rest, and functional reorganisation of network connections in ageing and dementia (Chan et al., 2014, Dipasquale et al., 2015, Geerligs et al., 2015, Grady et al., 2016, Sala-Llonch et al., 2015, Schouten et al., 2016, Smith et al., 2015).

Ageing is associated with domain-specific changes in cognitive ability, with declines in some but not other cognitive domains (Harada et al., 2013). The present study examined the resting-state functional connections associated with episodic and spatial memory using an unbiased data-driven network-modelling framework applied to 497 participants (60–82 years old) of the Whitehall II Imaging Sub-study. Given the vulnerability of these memory domains in ageing and AD, and task-fMRI evidence of their shared dependence on the hippocampus, we investigated whether, and how these two types of memory are related in terms of the underlying resting-state functional architecture supporting them. Specifically, we examined if associations between memory and resting-state functional connectivity were domain-specific and age-dependent.

## 2 Materials and methods

### 2.1 Participants

Participants belonged to the Whitehall II Imaging Sub-study, and the study protocol and MRI pre-processing pipeline has been described in detail previously (Filippini et al., 2014). Briefly, participants were drawn from the Whitehall II study, a cohort of 10 308 British Civil Servants established in University College London in 1985 and followed-up for over 30 years across 12 waves. For the Whitehall II Imaging Sub-study, 550 participants were randomly selected from the parent study; a battery of cognitive tests was administered followed by an MRI scan at the FMRIB Center, Oxford between 2012 and 2015. rs-fMRI data from 497 participants were used in this analysis. Exclusion criteria were incomplete or poor quality MRI data and/or structural abnormalities on the MRI scan (e.g. large tumours or brain cysts). Informed consent was obtained from all participants.

### 2.2 Verbal memory scores

Cognitive tests were administered by trained psychology graduates and psychiatrists and in the following order: Montreal Cognitive Assessment (MoCA), Trail Making Test (TMT-A and TMT-B), Lexical (letter: “F”) and Semantic Fluency (category: “Animals”), Rey-Osterrieth Complex Figure (ROCF) copying, RCF immediate recall, Hopkins Verbal Learning Test (HVLT-R) immediate recall, Boston Naming Test, Digit Span (forward, backward, ascending sequence) and Digit Coding (from the Wechsler Adult Intelligent Scale-IV), HVLT-R delayed recall, RCF delayed recall and Test of Premorbid Function (TOPF) (Filippini et al., 2014). All tests were included in a factor analysis (except the MoCA which is a screening tool for cognitive impairment). Factor analysis was performed using an oblique rotation (direct oblimin in SPSS 21) to allow for correlation between factors. The verbal memory factor (largest factor, accounting for ∼36% of the total variance) and visuospatial memory factor (accounting for ∼7% of the total variance) were used in this analysis. HVLT-R (immediate and delayed recall) and ROCF (copy, immediate and delayed recall) loaded high on verbal and visuospatial memory factors respectively (Table S1, Supplementary Materials). The HVLT-R is a list learning and free recall task comprising three trials of 12 words and is used to evaluate episodic verbal memory decline in dementia (Shapiro et al., 1999). The ROCF test involves copying a complex geometric figure and reproducing it from memory both immediately and following a delay, and is used to assess visuospatial memory and constructional ability (Shin et al., 2006).

### 2.3 MRI analysis

T1-weighted structural MRI (multi-echo MPRAGE sequence with motion correction) and multiband echo-planar imaging rs-fMRI scans (voxel = 2 mm isotropic, TR = 1.3 s, acquisition time = 10 min 10 s, multi-slice acceleration factor = 6, number of volumes = 460) were acquired. Participants were scanned on a 3T Siemens Magnetom Verio (Erlangen, Germany) scanner with a 32-channel head coil, at the FMRIB Center, Oxford. Data were pre-processed using FSL tools (Jenkinson et al., 2012, Smith et al., 2004) as described in Filippini et al., (2014). Network modelling was performed using FSLNets (Smith et al., 2013).

#### 2.3.1 Pre-processing and group-ICA

T1 images were bias field corrected, brain extracted and segmented into grey matter (GM), white matter (WM) and cerebrospinal fluid (CSF) using FSL-FAST. Rs-fMRI data were pre-processed (motion correction, brain extraction, high-pass temporal filtering at 100s, field-map correction) using FSL tools. FIX (FMRIB's ICA-based X-noisefier) was used to remove the artefactual components that reflected non-neuronal fluctuations (Griffanti et al., 2014, Salimi-Khorshidi et al., 2014). FIX was trained using the WhII_MB6.RData trained-weights file, which was generated from hand-labelling 25 participants from this study. This training file is available online (http://www.fmrib.ox.ac.uk/datasets/FIX-training/) and described, including the leave-one-out classification accuracy results, in Salimi-Khorshidi et al. (2014). The pre-processed and cleaned rs-fMRI scans were registered to standard space using FNIRT and spatially smoothed using a Gaussian kernel of 6 mm full width at half maximum. MELODIC group-ICA with a dimensionality of 100 was used to generate the group-level spatial maps (or *nodes*) for 500 participants who met the inclusion criteria. Small dimensionalities (<30) typically provide an estimate of whole resting-state networks whereas larger dimensionalities (>70) are used to define smaller nodes which can be used in network modelling analyses (Abou-Elseoud et al., 2009). As dimensionalities >100 show decreasing ICA repeatability, we chose 100 ICs, which is in line with recent studies adopting similar high-dimensionality ICA parcellations in multiband rsfMRI datasets (Miller et al., 2016, Smith et al., 2013). The resulting nodes were mapped onto each subject's rs-fMRI data to derive subject-specific time series (i.e. one time series per node for each subject) using the first stage of dual-regression (Beckmann et al., 2009, Filippini et al., 2009). Three subjects had incomplete cognitive tests and were excluded from subsequent network modelling analysis.

#### 2.3.2 Network modelling and cross-subject statistics

The time courses were fed into FSLNets (v0.6) to perform network modelling (Smith et al., 2013). Of the 100 nodes, 42 were discarded as noise components (white matter, physiological noise, MRI or movement artefacts) and the remaining 58 nodes were used to create the network matrix (*netmat* or *connectome*). A similar ratio of signal:noise (55:45) components was found in the 100-dimensional group-ICA from thousands of subjects in UK Biobank data (Miller et al., 2016).

The netmat is a Node x Node correlation matrix, with each matrix element representing the correlation strength (*edge* or *connectivity*) between the corresponding pair of nodes. To obtain a better estimate of the direct connections between nodes, we used partial correlation coefficients (with rho = 0.01 in Ridge Regression option in FSLNets) that were converted from Pearson correlation r-values into z-statistics with Fisher's transformation (Smith et al., 2011). The 58 nodes were reordered according to a hierarchical clustering of the group-average full correlation netmat using Ward's method implemented in Matlab to generate the functional connectome. Each subject's partial correlation netmat was then unwrapped into a single row and combined across subjects to create a Subject x Edges matrix, which represents all the participants' connectomes. Each column (*i.e.* a given edge, estimated for all subjects) in this matrix was correlated with verbal and visuospatial memory scores using a linear regression in FSLNets.

Given the complex interdependence between age and memory (Fjell et al., 2014, Nyberg et al., 2012), we performed two regressions in FSLNets to assess if the associations between memory and resting-state functional connectivity were (1) domain-specific and (2) age-dependent. First, to test domain specificity, we included verbal (or visuospatial) memory as an independent variable, and GM volume (normalized to total intracranial volume), average head motion during the scan, and visuospatial (or verbal) memory scores as confounding covariates in a GLM with the edges as dependent variables. Second, to assess if the observed rsFC – memory associations were age-dependent, we added age as a further covariate to the above model. The FSL randomize tool with 5 000 permutations was used to correct for multiple comparisons (controlling family-wise error, FWE) across all edges. Results that were significant at FWE-corrected p < 0.05 are reported. Linear regression (with usual diagnostic checks) was performed in SPSS 21 with verbal or visuospatial memory as the dependent variable and age as the independent variable.

## 3 Results

### 3.1 Socio-demographics and cognitive performance

Participants were 69.4 years old and the population characteristics reflect the demographics of the British Civil Service in 1985 at recruitment to the Whitehall II study; 80% of the participants in this study were male, with an average of 14.6 years of education. MoCA scores ranged from 17 to 30, and 145 participants (29.2% of sample) scored < 26. The HVLT-R and ROCF tests loaded highly on the verbal and visuospatial memory factors respectively (factor analysis displayed in Table S1, Supplementary Methods). Rotated factor scores were used in the analysis, but for descriptive purposes, the respective raw single test scores are shown below in Table 1.

**Table 1:** Population characteristics, brain measures and raw test scores for HVLT-R, ROCF and MoCA for 497 participants of the Whitehall II Imaging Sub-study. HVLT-R and ROCF loaded highly on the verbal and visuospatial memory factors respectively. Values represent mean ± standard deviation.

**Demographics**	
Age (years)	69.4 ± 5.2 (range: 60.3–82.0)
Sex (% male)	79.9%
Education (years)	14.6 ± 3.3
**Brain measures**	
Total brain volume (l)	1.4 ± 0.1
Grey matter (%)	38.5 ± 1.9
White matter (%)	38.8 ± 1.9
Cerebrospinal fluid (%)	22.7 ± 2.8
**Cognitive performance**	
MoCA score (points)	27.2 ± 2.3 (range: 17–30)
MoCA score (% below 26)	29.2%
HVLT-R Total recall (words)	27.7 ± 4.6 (range: 11–36)
HVLT-R Delayed recall (words)	9.3 ± 2.6 (range: 0–12)
ROCF Copy (score)	31.2 ± 4.0 (range: 6–36)
ROCF Immediate recall (score)	15.6 ± 6.6 (range 0–32)
ROCF Delayed recall (score)	15.3 ± 6.2 (range 0–30)

### 3.2 Functional connectome

**Fig. 1:**
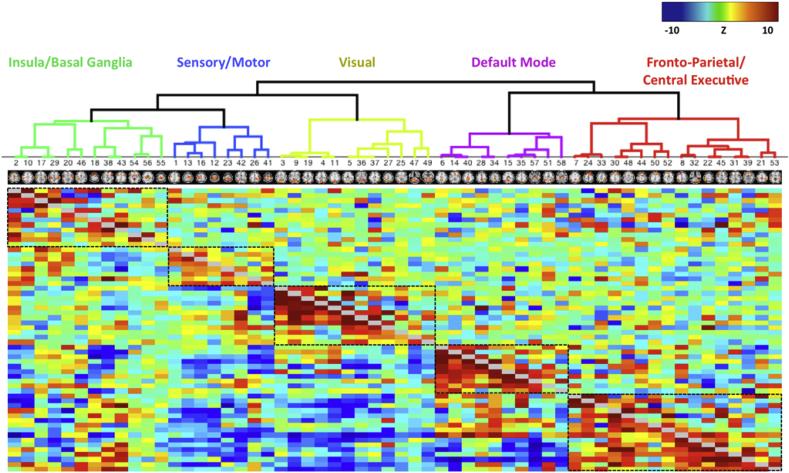
**The resting-state functional connectome estimated for 497 participants of the Whitehall II Imaging sub-study**. High-dimensionality group ICA and network modelling were performed using the FSL-MELODIC and FSLNETS tools respectively. Z statistics for the full correlation (below the diagonal) and partial correlation (above the diagonal) were computed for the 58 nodes visualized at the top of each column. The nodes were reordered according to a hierarchical clustering of the full correlation matrix. Five clusters representing commonly observed resting-state networks are highlighted in black boxes and labelled at the top of the figure. The partial correlation netmats were used in the linear regression with memory.

The functional connectivity matrix produced by FSLNets was estimated for 58 distinct nodes derived from 497 participants (Fig. 1). Because the correlation matrices are symmetrical, both full correlation (below the diagonal) and partial correlations (above the diagonal) between the corresponding node pairs are presented in the same matrix (only the ridge-regularised partial correlations were used in the regression against verbal and visuospatial memory). Groups of highly correlated nodes were clustered together according to a hierarchical clustering. The clusters were categorized into five large-scale networks (visualized at the top of the matrix) based on the spatial characteristics of their composite nodes, *viz.* the *insula/basal ganglia network* (central and parietal operculum cortex, planum polare and temporale, Heschl's gyrus, inferior frontal gyrus, thalamus, basal ganglia, precentral gyrus), *sensory/motor network* (precentral gyrus, postcentral gyrus, supplementary motor cortex, superior parietal lobule), *visual network* (medial and lateral visual cortices, cerebellum), *default mode network* (anterior and posterior cingulate cortex, precuneus, lateral temporal cortex, hippocampal formation), and the *fronto-parietal network/central executive network (CEN)* (frontal pole, superior and middle prefrontal cortices, parietal cortex, supramarginal gyrus).

### 3.3 Functional connection associated with verbal memory

**Fig. 2:**
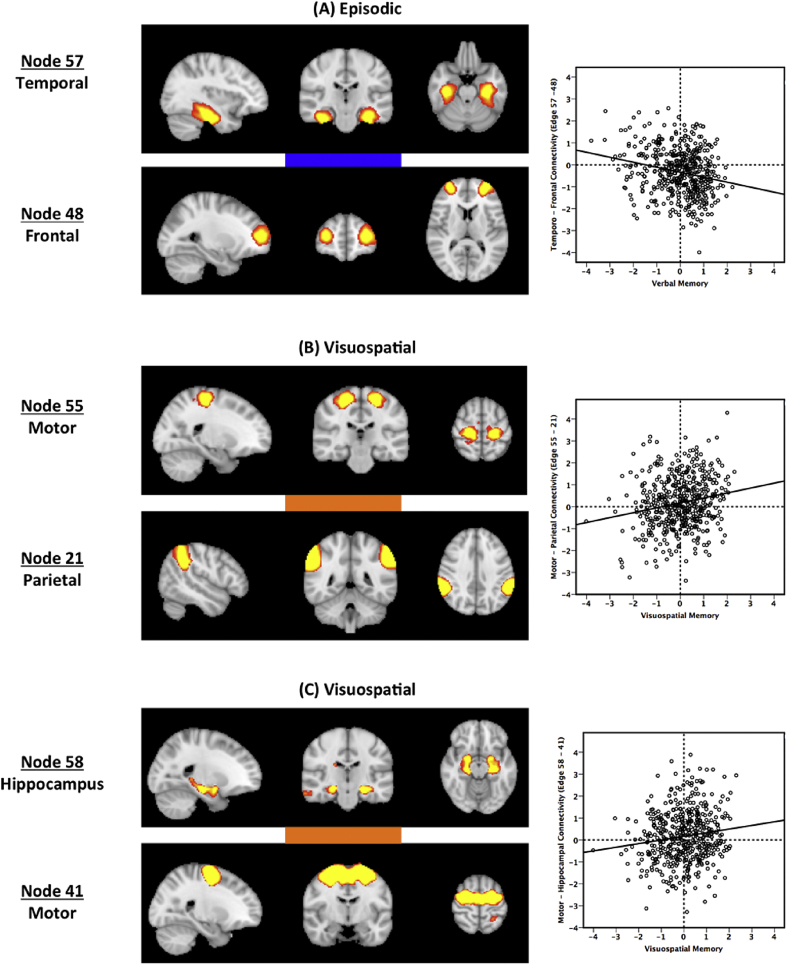
**Distinct resting-state connections representing episodic and visuospatial memory**. (a) Verbal memory was significantly negatively correlated with temporal-frontal anticorrelation. Visuospatial memory was significantly positively correlated with (b) motor-parietal connectivity and (c) motor-hippocampal connectivity. The colour of the bar connecting the two nodes represents the sign of the group-average partial correlation (red: positive, blue: negative). Each subject's partial correlation edge strengths are plotted against the corresponding memory factor on the right. All images are thresholded at z > 4 for visualisation.

One distinct connection, between the temporal node (node 57) and the frontal pole (node 48), was significantly negatively associated with verbal memory performance (FWE-corrected p < 0.05, correcting for grey matter volume, motion and visuospatial memory scores). The temporal node included the bilateral hippocampi, parahippocampal gyrus, and bilateral temporal fusiform cortex, and belonged to the cluster representing the DMN. The frontal pole node was part of the fronto-parietal/CEN cluster (Table 2). The two nodes showed a group-average anticorrelation, represented as a blue bar connecting the nodes in Fig. 2a (Z-statistic for group-average partial correlation = −7.02 for edge 57-48). Thus, greater temporo-frontal anticorrelation was uniquely associated with better verbal memory.

**Table 2:** Spatial details of the significant nodes.

Node	MNI coordinates of peak (x,y,z)	Location of peak	Node description	Network
21 “Parietal”	58, −42, 38	Right posterior supramarginal gyrus	Bilateral supramarginal gyrus	Fronto-parietal/CEN
41 “Motor”	24, −8, 62	Precentral gyrus	Supplementary motor cortex, precentral gyrus	Sensory/Motor
48 “Frontal”	−28, 56, 12	Frontal pole	Frontal pole	Fronto-parietal/CEN
55 “Motor”	18, −26, 60	Precentral gyrus	Precentral gyrus	Insula/Basal Ganglia
57 “Temporal”	−32, −20, −26	Left parahippocampal gyrus	Bilateral posterior temporal fusiform cortex, parahippocampal gyrus, posterior hippocampus	DMN
58 “Hippocampus”	−22, −20, −14	Left anterior hippocampus	Bilateral hippocampus, amygdala, parahippocamal gyrus	DMN

### 3.4 Functional connection associated with visuospatial memory

Two distinct edges were significantly associated with visuospatial memory performance (FWE-corrected p < 0.05, correcting for grey matter volume, motion, and verbal memory scores): (1) edge 58-41 between the hippocampus (node 58, part of the DMN) and motor cortex (node 41, part of the sensory/motor network), and (2) edge 55-21 between the precentral gyrus (node 55, part of the auditory/basal ganglia cluster) and bilateral supramarginal gyrus (node 21, part of the fronto-parietal/CEN cluster) (Table 2, Fig. 2b and c). The hippocampal node included the bilateral hippocampi, the bilateral amygdala, and parahippocampal gyri. Both edges had a group-average positive connection, represented as red bars connecting the nodes in Fig. 2b and c (Z = 3.13 and Z = 3.35 for edge 58-41 and 55-21 respectively). Thus, higher functional connectivity between edges 58-41 and 55-21 was uniquely associated with better visuospatial memory.

### 3.5 Contribution of age to resting-state functional connections associated with memory

Both verbal (R^2^ = 0.103, unstandardized B = −0.061 [95% CI: −0.077, −0.045], p < 0.001) and visuospatial memory performance (R^2^ = 0.044, unstandardized B = −0.04 [95% CI: −0.056, −0.023], p < 0.001) significantly declined with age. Edges 58-41 and 55-21 remained significantly and uniquely associated with visuospatial memory after age was added as a covariate to the regression model in FSLNets (FWE-corrected p < 0.05, correcting for grey matter volume, motion, verbal memory and age). By contrast, the association between edge 57-48 and verbal memory was no longer significant after correcting for age.

## 4 Discussion

This study presents a resting-state connectome for the Whitehall II Imaging Sub-study that maps the intrinsic functional organisation of the brain. Using a data-driven network modelling analysis, 58 distinct functional nodes generated from a high-dimensionality group-level ICA were organized into five large-scale networks reflecting auditory, somatosensory, motor and visual activity, as well as higher cognitive processes like memory, introspection and executive function. Notably, this organisation is largely consistent with the resting-state networks that are commonly identified using low-dimensionality ICA and have been consistently reported in healthy subjects (Damoiseaux et al., 2006) and across disease states (Baggio et al., 2015, Kim et al., 2015). Here, we identified distinct resting-state connections that were uniquely coupled with verbal and visuospatial memory in late adulthood. Anticorrelated activity between the temporal and frontal nodes was related to better verbal memory, whereas higher hippocampal-motor and parietal-motor functional connectivity was associated with better visuospatial memory. These associations were independent of grey matter volume and are thus unlikely to be driven by global age-related grey matter atrophy. Of note, unlike with visuospatial memory, the associations between verbal memory and the respective rsFCs were dependent on age (this is further discussed in Section 4.3).

### 4.1 Episodic and visuospatial memory share a common MTL substrate

Neuroimaging studies in healthy adults and AD patients report preferential engagement of the medial temporal lobe, particularly the hippocampus, in episodic memory tasks (Desgranges et al., 2002, Grady et al., 2003). Pioneering cognitive models suggest that the hippocampus plays a crucial role in spatial memory (*Cognitive Map Theory*, (O'Keefe and Nadel, 1978)) and episodic memory (*Multiple Trace Theory*, (Nadel and Moscovitch, 1997)), and that hippocampal involvement in spatial representations may underlie its role in episodic memory (Maguire and Mullally, 2013).

The resting-state functional interactions reported here provide support, in part, for all theories. We found that both verbal memory and visuospatial memory engaged the MTL nodes of the DMN (nodes 57 (temporal) and 58 (hippocampus) for verbal and visuospatial memory respectively). Previous graph-analytic approaches have suggested that the DMN is a heterogeneous network made up of two distinct sub-systems: a “dorsomedial prefrontal cortex subsystem” including the dorsomedial prefrontal cortex, temporoparietal junction, lateral temporal cortex, and an “MTL subsystem” including the parahippocampal cortex, hippocampal formation, ventromedial prefrontal cortex (Andrews-Hanna et al., 2010b). Task fMRI studies have reported a shared dependence of episodic and spatial memory specifically on the MTL subsystem of the DMN (Robin et al., 2015). Notably, we report a similar reliance of verbal and visuospatial memory on MTL areas at resting-state, without make any prior assumptions about the organisation of the DMN. The two MTL nodes in this study exhibited a degree of spatial overlap in the parahippocampal gyrus and the hippocampus, but were differently placed along the anterior-posterior axis. Visuospatial memory engaged the relatively anterior MTL node, including the amygdala, anterior parahippocampal gyrus and hippocampus while verbal memory decline was associated with the posterior parahippocampal gyrus, hippocampus, and temporal fusiform cortex. There are several competing theories on the long-axis functional specialisation of the hippocampal formation (Poppenk et al., 2013). Some volumetric and task-fMRI studies support the segregation of episodic memory and encoding of novel stimuli to the anterior hippocampus, and navigation/visuospatial memory and encoding of familiar stimuli to the posterior hippocampus (Hirshhorn et al., 2012, Nadel et al., 2013, Ryan et al., 2009). This view has been challenged by growing evidence for anterior hippocampal involvement in spatial representations, orientation, and encoding of novel spatial configurations (for reviews see (Poppenk et al., 2013, Zeidman and Maguire, 2016)). More research is needed to determine whether the two MTL nodes engaged here are spatially specific to each memory type (i.e. node 57 for verbal vs node 58 for visuospatial), and/or if they reflect a more general MTL-based mechanism supporting both verbal and visuospatial memory.

### 4.2 Domain-specific dissociations in MTL connectivity

While the MTL nodes of the DMN were related to visuospatial and verbal memory processes, we observed that the functional connections of MTL with other brain structures varied depending on the memory demand.

Visuospatial memory was associated with connectivity between the hippocampus and motor cortex, and between the supramarginal gyrus and precentral gyri. These *temporal-motor* and *parietal-motor* interactions are consistent with the cognitive abilities assessed by the copy and recall conditions of the ROCF test, *viz*. visual-motor function, executive skills, strategic planning and organisation, and visuospatial memory (Shin et al., 2006). By contrast, verbal episodic memory was associated with connectivity of the temporal fusiform/hippocampus and the frontal pole. Episodic memory is thought to rely on two interacting cognitive components, each with distinct neural correlates (Eichenbaum, 2009, Moscovitch, 1992), for review see (Shing et al., 2010). The *associative* component of episodic memory refers to the mechanisms that bind different features of an event into a cohesive memory episode, whereas the *strategic* component involves memory control operations like making use of existing semantic knowledge or internal/external cues to encode and retrieve memories (Shing et al., 2010). Animal models, lesion experiments, and human task-activation studies have inferred that the associative component of episodic memory relies on the MTL whereas the strategic component depends on the frontal and prefrontal cortices (Achim and Lepage, 2005, Moscovitch, 1992, Robin et al., 2015, Shing et al., 2010). Our finding is consistent with the two-component framework, and suggests that verbal memory in older adults may rely not only on discrete MTL and prefrontal processes, but also on the dynamic interaction between these regions and their functional organisation within the resting-state brain.

### 4.3 Domain-specific ageing effects

Age and cognitive performance are closely intertwined and it is arguably impossible to fully understand one without the other, particularly after mid-life when memory decline begins (Fjell et al., 2014, Nyberg et al., 2012, Singh-Manoux et al., 2012). Here, we observed a domain-specific dissociation in the dependence of the memory-rsFC relationship on age. Visuospatial memory was related to hippocampal-motor and parietal-motor connectivity in an age-independent manner, suggesting that these rsFCs may be on-going functional interactions that are continually present from young adulthood and maintained in older ages to support visuospatial memory. By contrast, age mediated the association between verbal memory and temporo-frontal anticorrelation. Collective evidence supports an early and preferential vulnerability of the episodic memory domain in ageing (Nyberg et al., 2012, Shing et al., 2010) and in our sample, age explained a greater proportion of total variation of verbal memory (∼10%) than visuospatial memory performance (∼4%). Ageing has also been associated with altered brain network dynamics between anti-correlated networks (Fox et al., 2005, Spreng et al., 2016). The temporo-frontal anticorrelation observed in this study likely reflects the functional specialisation of the parent large-scale networks (*i.e.* the DMN and CEN respectively). Anti-correlation between the DMN and CEN is considered a hallmark of the functional architecture of the resting brain and is essential for regulating brain activity (Fox et al., 2005, Nekovarova et al., 2014, Uddin et al., 2009). During cognitively challenging tasks, the DMN is typically deactivated whereas activity within the CEN is heightened. The two networks are also oppositely engaged during internally and externally directed cognitive tasks (Spreng et al., 2016) and this inverse pattern of activity between the DMN and CEN has been associated with better memory in younger adults (Hampson et al., 2010). Recent studies have reported age-associated reorganisation of this functional architecture, with reduced anti-correlation of the DMN and CEN in older relative to younger adults (Sala-Llonch et al., 2015, Spreng et al., 2016). In the context of emerging theories supporting dedifferentiation and reduced segregation of brain networks in ageing (Chan et al., 2014, Grady et al., 2016, Sala-Llonch et al., 2015, Spreng et al., 2016), we suggest that age-related decreases in the functional specialisation of the DMN and CEN may contribute to verbal episodic memory decline in older adulthood. Our results provide a more focused evaluation of this network segregation, and suggest that it is the strength of the anti-correlation between the temporal fusiform/hippocampal and frontal components of these two large-scale networks that may support verbal memory in older adults.

## 5 Summary

Cognitive decline in ageing is proposed to be a result of life-long accumulation of processes that impact brain structural, functional, metabolic, and pathological systems in a multi-dimensional way (Walhovd et al., 2014). With the rise in life expectancy, understanding the multi-system biological changes that support memory in older ages presents a timely challenge (Singh-Manoux et al., 2012). Our findings provide novel insights into the specific components of large-scale brain networks that relate to visuospatial and verbal episodic memory, in a relatively big sample with adequate statistical power to overcome inter-individual variability. Consistent with task-fMRI studies, we report a shared dependence of episodic and visuospatial memory on MTL nodes of the DMN in the resting-state brain. We propose that while the two memory types might rely on an MTL-associated mechanism, they differ in terms of the functional interactions of the MTL nodes; verbal memory recruits additional frontal areas, whereas visuospatial memory engages motor and parietal regions. Our results suggest an age-dependent mechanism for verbal memory decline that may rely on reduced anti-correlation between default mode and central executive networks. Longitudinal studies and studies across a wider age range will help clarify if age-related network dedifferentiation is directly related to verbal memory decline. Finally, we cannot make inferences about the directionality (node A→ node B or vice versa) of the connections reported here, and examinations of the structural connections between these nodes in healthy adults and patients with dementia would further our understanding of the complex role of the hippocampus in supporting these two types of memory.

## 6 Funding sources

Work on the Whitehall II Imaging Sub-study was mainly funded by the “Lifelong Health and Wellbeing” Programme Grant: “Predicting MRI abnormalities with longitudinal data of the Whitehall II Substudy” (UK Medical Research Council: G1001354). Sana Suri, NF, EZs, and AM are funded by the HDH Wills 1965 Charitable Trust (Nr: 1117747). AT is supported by UK Medical Research Council: (G1001354) and Oxford University Clinical Academic Graduate School. ASM receives research support from the US National Institutes of Health (R01AG013196, R01AG034454). MK is supported by the UK Medical Research Council (K013351) and NordForsk, the Nordic Programme on Health and Welfare. CEM and CES are supported by the National Institute for Health Research (NIHR) Oxford Health Biomedical Research Centre based at Oxford University Hospitals NHS Trust and University of Oxford. Stephen Smith is supported by the Wellcome Trust Strategic Award (098369/Z/12/Z). The Wellcome Centre for Integrative Neuroimaging is supported by core funding from the Wellcome Trust (203139/Z/16/Z). Authors do not report any conflict of interest. The study follows MRC data sharing policies [https://www.mrc.ac.uk/research/policies-and-guidance-for-researchers/data-sharing/]. Data will be accessible from the authors after 2019.

## References

[bib1] Abou-Elseoud A., Starck T., Remes J., Nikkinen J., Tervonen O., Kiviniemi V. (2009). The effect of model order selection in group PICA. Hum. Brain Mapp..

[bib2] Achim A.M., Lepage M. (2005). Dorsolateral prefrontal cortex involvement in memory post-retrieval monitoring revealed in both item and associative recognition tests. Neuroimage.

[bib3] Allen G., Barnard H., McColl R., Hester A.L., Fields J.A., Weiner M.F., Ringe W.K., Lipton A.M., Brooker M., McDonald E., Rubin C.D., Cullum C.M. (2007). Reduced hippocampal functional connectivity in Alzheimer disease. Arch. Neurol..

[bib4] Andrews-Hanna J.R., Reidler J.S., Huang C., Buckner R.L. (2010). Evidence for the default network's role in spontaneous cognition. J. Neurophysiol..

[bib5] Andrews-Hanna J.R., Reidler J.S., Sepulcre J., Poulin R., Buckner R.L. (2010). Functional-anatomic fractionation of the Brain's default network. Neuron.

[bib6] Andrews-Hanna J.R., Snyder A.Z., Vincent J.L., Lustig C., Head D., Raichle M.E., Buckner R.L. (2007). Disruption of large-scale brain systems in advanced aging. Neuron.

[bib7] Baggio H.-C., Segura B., Sala-Llonch R., Marti M.-J., Valldeoriola F., Compta Y., Tolosa E., Junqué C. (2015). Cognitive impairment and resting-state network connectivity in Parkinson's disease. Hum. Brain Mapp..

[bib8] Beckmann, Mackay, Filippini, Smith (2009). Group comparison of resting-state FMRI data using multi-subject ICA and dual regression. Neuroimage.

[bib9] Buckner R.L. (2010). The role of the Hippocampus in prediction and imagination. Annu. Rev. Psychol..

[bib10] Buckner R.L., Andrews-Hanna J.R., Schacter D.L. (2008). The brain's default network: anatomy, function, and relevance to disease. Ann. N. Y. Acad. Sci..

[bib11] Chan M.Y., Park D.C., Savalia N.K., Petersen S.E., Wig G.S. (2014). Decreased segregation of brain systems across the healthy adult lifespan. Proc. Natl. Acad. Sci..

[bib12] Cole D.M., Smith S.M., Beckmann C.F. (2010). Advances and pitfalls in the analysis and interpretation of resting-state FMRI data. Front. Syst. Neurosci..

[bib13] Damoiseaux J.S., Beckmann C.F., Arigita E.J.S., Barkhof F., Scheltens P., Stam C.J., Smith S.M., Rombouts S.A.R.B. (2008). Reduced resting-state brain activity in the “default network” in normal aging. Cereb. Cortex.

[bib14] Damoiseaux J.S., Rombouts S.A.R.B., Barkhof F., Scheltens P., Stam C.J., Smith S.M., Beckmann C.F. (2006). Consistent resting-state networks across healthy subjects. Proc. Natl. Acad. Sci. U. S. A..

[bib15] Delbeuck X., Van der Linden M., Collette F. (2003). Alzheimer's disease as a disconnection syndrome?. Neuropsychol. Rev..

[bib16] Desgranges B., Baron J., Lalevée C., Giffard B., Viader F., de la Sayette V., Eustache F. (2002). The neural substrates of episodic memory impairment in Alzheimer's disease as revealed by FDG–PET: relationship to degree of deterioration. Brain.

[bib17] Dipasquale O., Griffanti L., Clerici M., Nemni R., Baselli G., Baglio F. (2015). High-dimensional ICA analysis detects within-network functional connectivity damage of default-mode and sensory-motor networks in Alzheimerâ€™s disease. Front. Hum. Neurosci..

[bib18] Eichenbaum, H., 2009. The cognitive neuroscience of memory: an introduction, the cognitive neuroscience of memory: an introduction.10.1093/acprof:oso/9780195141740.001.0001.

[bib19] Ferreira L.K., Busatto G.F. (2013). Resting-state functional connectivity in normal brain aging. Neurosci. Biobehav. Rev..

[bib20] Filippini N., MacIntosh B.J., Hough M.G., Goodwin G.M., Frisoni G.B., Smith S.M., Matthews P.M., Beckmann C.F., Mackay C.E. (2009). Distinct patterns of brain activity in young carriers of the APOE-epsilon4 allele. Proc. Natl. Acad. Sci. U. S. A..

[bib21] Filippini N., Zsoldos E., Haapakoski R., Sexton C.E., Mahmood A., Allan C.L., Topiwala A., Valkanova V., Brunner E.J., Shipley M.J., Auerbach E., Moeller S., Uğurbil K., Xu J., Yacoub E., Andersson J., Bijsterbosch J., Clare S., Griffanti L., Hess A.T., Jenkinson M., Miller K.L., Salimi-Khorshidi G., Sotiropoulos S.N., Voets N.L., Smith S.M., Geddes J.R., Singh-Manoux A., Mackay C.E., Kivimäki M., Ebmeier K.P. (2014). Study protocol: the Whitehall II imaging sub-study. BMC Psychiatry.

[bib22] Fjell A.M., McEvoy L., Holland D., Dale A.M., Walhovd K.B. (2014). What is normal in normal aging? Effects of aging, amyloid and Alzheimer's disease on the cerebral cortex and the hippocampus. Prog. Neurobiol..

[bib23] Fox M.D., Snyder A.Z., Vincent J.L., Corbetta M., Van Essen D.C., Raichle M.E. (2005). The human brain is intrinsically organized into dynamic, anticorrelated functional networks. Proc. Natl. Acad. Sci. U. S. A..

[bib24] Geerligs L., Renken R.J., Saliasi E., Maurits N.M., Lorist M.M. (2015). A brain-wide study of age-related changes in functional connectivity. Cereb. Cortex.

[bib25] Grady C., Sarraf S., Saverino C., Campbell K. (2016). Age differences in the functional interactions among the default, frontoparietal control, and dorsal attention networks. Neurobiol. Aging.

[bib26] Grady C.L., McIntosh A.R., Beig S., Keightley M.L., Burian H., Black S.E. (2003). Evidence from functional neuroimaging of a compensatory prefrontal network in Alzheimer's disease. J. Neurosci..

[bib27] Griffanti L., Salimi-Khorshidi G., Beckmann C.F., Auerbach E.J., Douaud G., Sexton C.E., Zsoldos E., Ebmeier K.P., Filippini N., Mackay C.E., Moeller S., Xu J., Yacoub E., Baselli G., Ugurbil K., Miller K.L., Smith S.M. (2014). ICA-based artefact removal and accelerated fMRI acquisition for improved resting state network imaging. Neuroimage.

[bib28] Hafkemeijer A., van der Grond J., Rombouts S.A.R.B. (2012). Imaging the default mode network in aging and dementia. Biochim. Biophys. Acta - Mol. Basis Dis..

[bib29] Hampson M., Driesen N., Roth J.K., Gore J.C., Constable R.T. (2010). Functional connectivity between task-positive and task-negative brain areas and its relation to working memory performance. Magn. Reson. Imaging.

[bib74] Harada C.N., Natelson Love M.C., Triebel K.L. (2013). Normal cognitive aging. Clin. Geriatr. Med..

[bib30] Hirshhorn M., Grady C., Rosenbaum R.S., Winocur G., Moscovitch M. (2012). Brain regions involved in the retrieval of spatial and episodic details associated with a familiar environment: an fMRI study. Neuropsychologia.

[bib31] Huijbers W., Pennartz C.M.A., Cabeza R., Daselaar S.M. (2011). The Hippocampus is coupled with the default network during memory retrieval but not during memory encoding. PLoS One.

[bib32] Jack C.R., Shiung M.M., Gunter J.L., O'Brien P.C., Weigand S.D., Knopman D.S., Boeve B.F., Ivnik R.J., Smith G.E., Cha R.H., Tangalos E.G., Petersen R.C. (2004). Comparison of different MRI brain atrophy rate measures with clinical disease progression in AD. Neurology.

[bib33] Jenkinson M., Beckmann C., Behrens T.E., Woolrich M.W., Smith S.M. (2012). FSL. Neuroimage.

[bib34] Kim H.J., Cha J., Lee J.-M., Shin J.S., Jung N.-Y., Kim Y.J., Choe Y.S., Lee K.H., Kim S.T., Kim J.S., Lee J.H., Na D.L., Seo S.W. (2015). Distinctive resting state network disruptions among Alzheimer's disease, subcortical vascular dementia, and mixed dementia patients. J. Alzheimers. Dis..

[bib35] Kumaran D., Maguire E.A. (2005). The human Hippocampus: cognitive maps or relational memory?. J. Neurosci..

[bib36] Lange K.L., Bondi M.W., Salmon D.P., Galasko D., Delis D.C., Thomas R.G., Thal L.J. (2002). Decline in verbal memory during preclinical Alzheimer's disease: examination of the effect of APOE genotype. J. Int. Neuropsychol. Soc..

[bib37] Maguire E.A., Mullally S.L. (2013). The hippocampus: a manifesto for change. J. Exp. Psychol. Gen..

[bib38] Mevel K., Chételat G., Eustache F., Desgranges B., Mevel K., Chételat G., Eustache F., Desgranges B., Ché, telat G.L., Eustache F., Desgranges B., Atrice (2011). The default mode network in healthy aging and Alzheimer's disease. Int. J. Alzheimers. Dis..

[bib39] Miller K.L., Alfaro-Almagro F., Bangerter N.K., Thomas D.L., Yacoub E., Xu J., Bartsch A.J., Jbabdi S., Sotiropoulos S.N., Andersson J.L.R., Griffanti L., Douaud G., Okell T.W., Weale P., Dragonu I., Garratt S., Hudson S., Collins R., Jenkinson M., Matthews P.M., Smith S.M. (2016). Multimodal population brain imaging in the UK Biobank prospective epidemiological study. Nat. Neurosci..

[bib40] Moscovitch M. (1992). Memory and working-with-memory: a component process model based on modules and central systems. J. Cogn. Neurosci..

[bib41] Nadel L., Hoscheidt S., Ryan L.R. (2013). Spatial cognition and the hippocampus: the anterior-posterior axis. J. Cogn. Neurosci..

[bib42] Nadel L., Moscovitch M. (1997). Memory consolidation, retrograde amnesia and the hippocampal complex. Curr. Opin. Neurobiol..

[bib43] Nekovarova T., Fajnerova I., Horacek J., Spaniel F. (2014). Bridging disparate symptoms of schizophrenia: a triple network dysfunction theory. Front. Behav. Neurosci..

[bib44] Nyberg L., Lovden M., Riklund K., Lindenberger U., Backman L. (2012). Memory aging and brain maintenance. Trends Cogn. Sci..

[bib45] O'Keefe J., Nadel L. (1978). The Hippocampus as a Cognitive Map.

[bib46] Onoda K., Ishihara M., Yamaguchi S. (2012). Decreased functional connectivity by aging is associated with cognitive decline. J. Cogn. Neurosci..

[bib47] Poppenk J., Evensmoen H.R., Moscovitch M., Nadel L. (2013). Long-axis specialization of the human hippocampus. Trends Cogn. Sci..

[bib48] Raichle M.E., MacLeod A.M., Snyder A.Z., Powers W.J., Gusnard D.A., Shulman G.L. (2001). A default mode of brain function. Proc. Natl. Acad. Sci. U. S. A..

[bib49] Robin J., Hirshhorn M., Rosenbaum R.S., Winocur G., Moscovitch M., Grady C.L. (2015). Functional connectivity of hippocampal and prefrontal networks during episodic and spatial memory based on real-world environments. Hippocampus.

[bib50] Ryan L., Lin C.-Y., Ketcham K., Nadel L. (2009). The role of medial temporal lobe in retrieving spatial and nonspatial relations from episodic and semantic memory. Hippocampus.

[bib51] Sala-Llonch R., Bartrés-Faz D., Junqué C. (2015). Reorganization of brain networks in aging: a review of functional connectivity studies. Front. Psychol..

[bib52] Salimi-Khorshidi G., Douaud G., Beckmann C.F., Glasser M.F., Griffanti L., Smith S.M. (2014). Automatic denoising of functional MRI data: combining independent component analysis and hierarchical fusion of classifiers. Neuroimage.

[bib53] Schouten T.M., Koini M., de Vos F., Seiler S., van der Grond J., Lechner A., Hafkemeijer A., Möller C., Schmidt R., de Rooij M., Rombouts S.A.R.B. (2016). Combining anatomical, diffusion, and resting state functional magnetic resonance imaging for individual classification of mild and moderate Alzheimer's disease. NeuroImage Clin..

[bib54] Serino S., Morganti F., Di Stefano F., Riva G. (2015). Detecting early egocentric and allocentric impairments deficits in Alzheimer's disease: an experimental study with virtual reality. Front. Aging Neurosci..

[bib55] Serino S., Riva G. (2014). What is the role of spatial processing in the decline of episodic memory in Alzheimer's disease? The “mental frame syncing” hypothesis. Front. Aging Neurosci..

[bib56] Shapiro A.M., Benedict R.H., Schretlen D., Brandt J. (1999). Construct and concurrent validity of the Hopkins verbal learning test-revised. Clin. Neuropsychol..

[bib57] Shin M.-S., Park S.-Y., Park S.-R., Seol S.-H., Kwon J.S. (2006). Clinical and empirical applications of the rey–osterrieth complex figure test. Nat. Protoc..

[bib58] Shing Y.L., Werkle-Bergner M., Brehmer Y., Müller V., Li S.-C., Lindenberger U. (2010). Episodic memory across the lifespan: the contributions of associative and strategic components. Neurosci. Biobehav. Rev..

[bib59] Singh-Manoux A., Kivimaki M., Glymour M.M., Elbaz A., Berr C., Ebmeier K.P., Ferrie J.E., Dugravot A. (2012). Timing of onset of cognitive decline: results from Whitehall II prospective cohort study. BMJ.

[bib60] Smith S.M., Jenkinson M., Woolrich M.W., Beckmann C.F., Behrens T.E.J., Johansen-Berg H., Bannister P.R., De Luca M., Drobnjak I., Flitney D.E., Niazy R.K., Saunders J., Vickers J., Zhang Y., De Stefano N., Brady J.M., Matthews P.M. (2004). Advances in functional and structural MR image analysis and implementation as FSL. Neuroimage.

[bib61] Smith S.M., Miller K.L., Salimi-Khorshidi G., Webster M., Beckmann C.F., Nichols T.E., Ramsey J.D., Woolrich M.W. (2011). Network modelling methods for FMRI. Neuroimage.

[bib62] Smith S.M., Nichols T.E., Vidaurre D., Winkler A.M., J Behrens T.E., Glasser M.F., Ugurbil K., Barch D.M., Van Essen D.C., Miller K.L. (2015). A positive-negative mode of population covariation links brain connectivity, demographics and behavior. Nat. Neurosci..

[bib63] Smith S.M., Vidaurre D., Beckmann C.F., Glasser M.F., Jenkinson M., Miller K.L., Nichols T.E., Robinson E.C., Salimi-Khorshidi G., Woolrich M.W., Barch D.M., Uğurbil K., Van Essen D.C. (2013). Functional connectomics from resting-state fMRI. Trends Cogn. Sci..

[bib64] Spreng R.N., Mar R.A., Kim A.S.N. (2009). The common neural basis of autobiographical memory, prospection, navigation, theory of mind, and the default mode: a quantitative meta-analysis. J. Cogn. Neurosci..

[bib65] Spreng R.N., Stevens W.D., Chamberlain J.P., Gilmore A.W., Schacter D.L. (2010). Default network activity, coupled with the frontoparietal control network, supports goal-directed cognition. Neuroimage.

[bib66] Spreng R.N., Stevens W.D., Viviano J.D., Schacter D.L. (2016). Attenuated anticorrelation between the default and dorsal attention networks with aging: evidence from task and rest. Neurobiol. Aging.

[bib67] Sridharan D., Levitin D.J., Menon V. (2008). A critical role for the right fronto-insular cortex in switching between central-executive and default-mode networks. Proc. Natl. Acad. Sci. U. S. A..

[bib68] Uddin L.Q., Kelly A.M.C., Biswal B.B., Castellanos F.X., Milham M.P. (2009). Functional connectivity of default mode network components: correlation, anticorrelation, and causality. Hum. Brain Mapp..

[bib69] van den Heuvel M.P., Sporns O. (2013). Network hubs in the human brain. Trends Cogn. Sci..

[bib70] Walhovd K.B., Fjell A.M., Espeseth T. (2014). Cognitive decline and brain pathology in aging - need for a dimensional, lifespan and systems vulnerability view. Scand. J. Psychol..

[bib71] WPA (2015). World Population Ageing 2015.

[bib72] Wu X., Li R., Fleisher A.S., Reiman E.M., Guan X., Zhang Y., Chen K., Yao L. (2011). Altered default mode network connectivity in Alzheimer's disease–a resting functional MRI and Bayesian network study. Hum. Brain Mapp..

[bib73] Zeidman P., Maguire E.A. (2016). Anterior hippocampus: the anatomy of perception, imagination and episodic memory. Nat. Rev. Neurosci..

