# Supplementary material for: Distinct resting-state functional connections associated with episodic and visuospatial memory in older adults
Source: Neuroimage. 2017 Oct 1;159:122–30. doi: 10.1016/j.neuroimage.2017.07.049 (PMC5678287; doi:10.1016/j.neuroimage.2017.07.049)
Supplement: Supplementary file 1 [file mmc1.docx]

**Supplementary Methods**

Table S1: Rotated structure matrix for factor analysis (direct oblimin). Five factors were identified. HVLT-R loaded high on the verbal memory factor (largest factor, used in this analysis). Abbreviations: HVLT-R: Hopkins Verbal Learning Test, ROCF: Rey-Osterrieth Complex Figure, TOPF: Test of Premorbid Function, BNT: Boston Naming Test; TMT: Trail Making Test.

|  | **1**  **Verbal Memory** | **2**  **Working Memory** | **3**  **Executive Function** | **4**  **Visuospatial Memory** | **5**  **General Intelligence** |
| --- | --- | --- | --- | --- | --- |
| HVLT-R (delayed) | .86 | .28 | -.33 | .31 | .18 |
| HVLT-R (total) | .86 | .35 | -.31 | .30 | .18 |
| Semantic fluency | .65 | .24 | -.35 | .25 | .38 |
| Digit span (total) | .38 | .99 | -.40 | .15 | .27 |
| Digit span (backward) | .29 | .85 | -.30 | .08 | .24 |
| Digit span (forward) | .16 | .81 | -.22 | .17 | .27 |
| Digit span (sequence) | .47 | .72 | -.44 | .11 | .14 |
| Trail Making Test B | -.28 | -.33 | .97 | -.31 | -.31 |
| TMT B – TMT A | -.16 | -.26 | .88 | -.25 | -.34 |
| Digit coding | .47 | .34 | -.71 | .25 | .15 |
| Trail Making Test A | -.44 | -.35 | .69 | -.30 | -.05 |
| ROCF delayed | .33 | .17 | -.34 | .94 | .19 |
| ROCF immediate | .32 | .14 | -.30 | .94 | .18 |
| ROCF copy | .23 | .24 | -.35 | .62 | .41 |
| TOPF | .19 | .37 | -.26 | .24 | .83 |
| BNT | .36 | .19 | -.36 | .33 | .78 |
| Letter fluency | .40 | .41 | -.26 | -.05 | .43 |
| % Explained variance of each factor | **35.9%** | **12.1 %** | **8.6 %** | **7.3%** | **6.5 %** |
